# Spatial Distribution and Pathogen Profile of *Dermacentor reticulatus* Ticks in Southeastern Poland: A Genetic and Environmental Analysis

**DOI:** 10.1155/2024/5458278

**Published:** 2024-04-24

**Authors:** Zbigniew Zając, Joanna Kulisz, Aneta Woźniak, Dasiel Obregón, Angélique Foucault-Simonin, Katarzyna Bartosik, Sara Moutailler, Alejandro Cabezas-Cruz

**Affiliations:** ^1^Department of Biology and Parasitology, Medical University of Lublin, Radziwiłłowska 11, 20-080, Lublin, Poland; ^2^School of Environmental Sciences, University of Guelph, Guelph, ON, N1G 2W1, Canada; ^3^Anses, INRAE, Ecole Nationale Vétérinaire d'Alfort, UMR BIPAR, Laboratoire de Santé Animale, 94700, Maisons-Alfort, France

## Abstract

In recent years, significant changes have been observed in the distribution and abundance of local *Dermacentor reticulatus* populations. However, changes in *D. reticulatus* dynamics have not been studied in southeastern Poland. Our objective was to enhance our understanding of the environmental factors influencing the occurrence and density of *D. reticulatus* in this area. Additionally, we sought to investigate the genetic diversity of the tick population and the prevalence of tick-borne pathogens (TBPs). To this end, we established 45 study sites in the Subcarpathian province. Ticks were collected during their peak activity in both spring and autumn. A subset of randomly selected specimens underwent molecular analysis for TBPs screening, using high-throughput microfluidic real-time PCR. Positive amplicons were then sequenced, and phylogenetic analyses were conducted. Our findings confirmed the presence of *D. reticulatus* ticks in 24 surveyed sites, primarily concentrated in the northern and eastern parts of the region. The mean density of *D. reticulatus* ticks in their compact range was 5.8 ± 6.4 specimens/100 m^2^. Notably, air temperature and altitude emerged as significant factors influencing the species' activity. We also identified a high prevalence of *Rickettsia raoultii* infections in adult *D. reticulatus*, reaching up to 84.21%. Additionally, 9.52% of ticks were found to be infected with *R. helvetica* and 4.76% with *Anaplasma phagocytophilum*. Furthermore, our genetic analyses confirmed the identity of *D. reticulatus* in the Subcarpathian region, aligning with haplotypes found in other regions of Poland, Czechia, Croatia, and Portugal. In conclusion, our study suggests that the surveyed region represents the current boundary of the compact range of *D. reticulatus* in Poland in which this tick species exhibits low genetic diversity and a narrow spectrum of detected TBPs.

## 1. Introduction

*Dermacentor reticulatus* ticks, widely distributed and abundant tick species across Europe, play a crucial role as vectors and reservoirs for tick-borne pathogens (TBPs) [1, 2]. This species demonstrates a remarkable ability to survive in diverse environmental conditions, supported by a broad range of potential hosts [3, 4]. Notably, over 40 species of TBPs have been identified in *D. reticulatus* ticks, posing a significant threat to animal and human health [1]. These ticks are capable of transmitting viruses such as *Orthoflavivirus encephalitidis*, the causative agent of tick-borne encephalitis, previously known as tick-borne encephalitis virus (TBEV) and Omsk hemorrhagic fever virus (OHFV) [1, 5, 6]. *D. reticulatus* ticks also play a significant role in the transmission of spotted fever rickettsiae group (SFRG), including *Rickettsia slovaca* and *R. raoultii* that may lead to lymphadenopathy (TIBOLA) and scalp eschar neck lymphadenopathy (SENLAT). By feeding on human skin, *D. reticulatus* can also lead to *Dermacentor*-borne-necrosis-erythema lymphadenopathy (DEBONEL) [1]. While the full extent of *D. reticulatus* competence as a vector for *Borrelia* spp., *Francisella tularensis*, and *Coxiella burnetii* is not yet fully understood, the genetic material of these bacteria is frequently detected in individuals of this tick species [1, 7, 8].

From a veterinary perspective, *D. reticulatus* ticks serve as the important vector for the piroplasmid apicomplexan parasite *Babesia canis*, causing canine babesiosis [9, 10]. Additionally, they transmit *B. caballi* and *Theileria equi*, leading to the most prevalent tick-borne disease in equids, namely piroplasmosis, and *Anaplasma marginale*, causing bovine anaplasmosis, the most important tick-borne disease of domesticated ruminants globally [11, 12].

In recent years, significant changes have been observed in the distribution of *D. reticulatus*, both at the regional level and across the European continent [3, 13]. Until the early 2000s, it was widely believed that there were two geographically separated populations of *D. reticulatus* in Europe. The Eastern European population covered areas east of the Vistula River line in Poland, extending into Slovakia, Hungary, and Romania, and reaching the steppes of Kazakhstan. In contrast, the Western European population was thought to encompass France, the Benelux countries, and Western Germany [14]. The areas between these regions were considered free of *D. reticulatus* [15]. However, the current scenario reveals a dynamic expansion of *D. reticulatus* into new areas. Recent reports confirm the presence of this tick species in the British Isles, the Mediterranean, and the Baltic regions [16–18]. The traditional border between the Eastern and Western European populations is becoming less distinct, with numerous new sites reported in Slovakia, Czechia, Hungary, Germany, and Romania [19–23]. Of particular interest is the situation in Poland, where in the eastern part of the country, a highly abundant population of *D. reticulatus* with a compact range is observed [24, 25], while in the western and central parts, a regular expansion of the range of this species is in progress [26–28]. The changes in tick distribution are primarily attributed to progressive climate change and its associated consequences [29, 30].

The increased prevalence of transmitted pathogens and the evolving distribution of *D. reticulatus* over the past two decades have sparked heightened attention and extensive research aimed at understanding the reasons for the observed changes. Our study aims to contribute to the knowledge of the environmental factors influencing the occurrence and density of *D. reticulatus* in southeastern Poland, specifically the Subcarpathian region. We hypothesized that the current range limit of this tick species may extend in the studied region. Additionally, given the potential co-occurrence of eastern and western European subpopulations in this area, our research also seeks to investigate their genetic diversity. Furthermore, we aim to examine the spectrum of TBPs vectored by *D. reticulatus* in the Subcarpathian region.

## 2. Materials and Methods

### 2.1. Study Area

**Figure 1:**
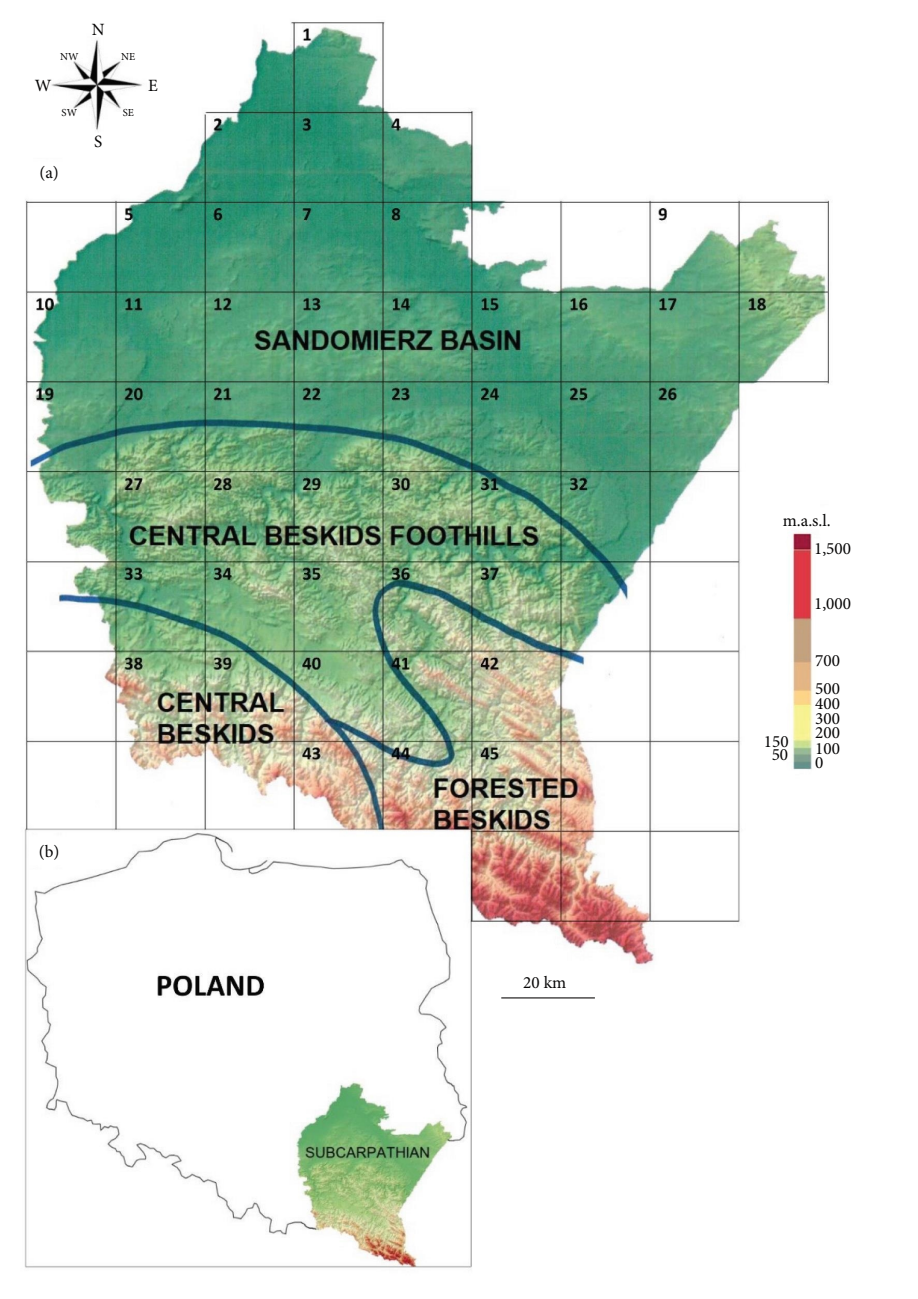
Overview map of the Subcarpathian province (Poland) including subregions and grid within which tick collection sites were established (a) against the background of Poland (b). This figure was generated using an online tool https://opentopomap.org and edited in GIMP 2.20.32 software (GIMP Development Team, https://www.gimp.org/).

The investigation focused on the density and occurrence of *D. reticulatus* ticks in southeastern Poland, covering the entire territory of the Subcarpathian province. Encompassing 17,846 km^2^, this region represents 5.70% of the country's total area. Notably diverse in geomorphology, climate, and potential vegetation, the studied area consists of smaller subregions: Central Beskids, Forested Beskids, Central Beskids Foothills, and Sandomierz Basin (Figure 1).

The Central Beskids and Forested Beskids subregions belong to the Carpathian Mountains, one of the largest mountain ranges in Europe. The prevailing climate varies with altitude, slope exposure, and the density of the valley network and is characterized by continental features. The average annual temperature ranges from 2.0 to 4.0°C over 1,000 m.a.s.l., while lower elevations record an average temperature of 6.3°C (averaged from 1950 to 2010), rising to 8.5°C in the last decade. Total rainfall is 885 mm a year. On average, maximum air temperature is below 0.0°C for 58 days per year, and snow covers 93 days. The dominant potential vegetation in the Central Beskids is the Carpathian beech forest Dentario glandulosae-Fagetum, syn. Fagetum carpaticum, mixed with subcontinental oak-hornbeam of the Carpathian variant Carici pilosae-Carpinetum betuli. Grassland communities dominate forests above 1,000 m.a.s.l. in the Forested Beskids subregion [31–33].

The Central Beskids Foothills subregion has a milder climate, with an average temperature of 8.9°C and an average annual rainfall of 771 mm. The maximum air temperature does not exceed 0.0°C for 40 days a year, while snow cover persists for 73 days. Tilio-Carpinetum, a subcontinental oak-hornbeam, is the dominant potential vegetation type throughout the subregion, with an island mosaic of Querco-Fagetea oak and Fagetum carpaticum Carpathian beech [31–33].

Geomorphologically, the Sandomierz Basin is part of pre-mountain tectonic depressions. Characterized as one of the warmest areas in Poland, it boasts a mean annual air temperature of 10.4°C. Over the past decade, the maximum air temperature below 0°C lasted for 27 days on average, with annual precipitation totaling 551 mm. Intensively used for agriculture, the landscape is dominated by arable land, and the potential vegetation is Tilio-Carpinetum, a subcontinental oak-hornbeam [31–33].

### 2.2. Establishment of Field Study Sites

To create field study sites, a 20 km side-length grid was overlaid onto the Subcarpathian province map, resulting in 45 squares, each covering 400 km^2^. Squares along the province border varied in size (Figure 1).

Subsequently, using a high-resolution orthophoto map from Geoportal [34], three potential tick collection sites were identified within each designated square. Preference was given to habitats recognized as preferred by *D. reticulatus* ticks, specifically, grasslands in progressive ecological succession, ideally located near the forest (Figure 1) [25, 35].

Field inspections were conducted at the predetermined tick collection sites. If ticks were not found in a particular site, the procedure was repeated at two additional sites within the same grid square. A grid square was deemed free of *D. reticulatus* if no ticks were confirmed at any of the selected sites.

### 2.3. Tick Surveillance

To investigate the seasonal activity of *D. reticulatus*, field studies were conducted during the peak periods of autumn (mid-October 2022) and spring (mid-April 2023) in eastern Poland. The rhythms of tick activity were determined based on our prior extensive studies on the species [4, 25, 35–37].

Ticks were collected from the same transects of 500 m^2^ during both autumn and spring surveys. A detailed description of the tick collection procedure can be found elsewhere [4, 24]. Additionally, a Data Logger R6030 device (R6030, Reed Instruments, Wilmington, NC, USA) was used to measure the actual air temperature and humidity at the vegetation level.

In the laboratory, collected specimens were meticulously identified by species, sex, and developmental stage using a Zeiss STEMI DV4 stereo microscope (Carl Zeiss Light Microscopy, Göttingen, Germany) and a taxonomic identification key [2]. Subsequently, the specimens were preserved frozen at −80°C (Arctico, Esbjerg, Denmark) until DNA extraction.

## 3. Molecular Analysis

### 3.1. DNA Extraction

In preparation for molecular analyses, previously collected ticks were washed with ultrapure water and dried. Next, specimens were cut into smaller fragments using a sterile scalpel. Tick DNA was extracted using the Genomic Mini AX Tissue kit (A&A Biotechnology, Gdynia, Poland) following the manufacturer's instructions. A NanoDrop 2000 spectrophotometer (Thermo Scientific, Waltham, USA) operating at 260/280 nm wavelength was used to quantify the concentration of extracted DNA. A total volume of 35 *µ*L elution provided isolates with a DNA concentration of 10–80 ng/*µ*L. Next, the samples were stored at −20°C until further processing.

### 3.2. DNA Preamplification

DNA preamplification was performed according to the manufacturer's protocol using the PreAmp Master Mix kit (Standard Biotools, San Francisco, CA, USA). We used the same procedure described in detail elsewhere [38, 39].

### 3.3. Microfluidic Real-Time PCR for High-Throughput Detection of Microorganisms

The BioMark™ real-time PCR system (Standard Biotools, San Francisco, USA) was used to detect the pathogens. Real-time PCR reactions were performed according to the manufacturer's protocol (Applied Biosystems, France) using 6-carboxyfluorescein (FAM)-labeled and black hole quencher (BHQ1)-labeled TaqMan probes with TaqMan Gene expression master mix. The reaction was carried out in the following steps: 2 min at 50°C, 10 min at 95°C, followed by 40 cycles of two-step amplification of 15 s at 95°C, and 1 min at 60°C.

A primer/probe set was used to confirm the identification of pathogens and tick species. To check for potential inhibition, an *E. coli*-specific primer/probe set was employed. In the real-time PCR reaction, we used the same primers as in a previously published paper [40] (Table S1). The negative control was ultrapure water.

The obtained results were analyzed using Standard Biotools Real-time PCR Analysis Software to calculate crossing threshold (Ct) values.

### 3.4. Validation of Microfluidic Real-Time PCR

In order to validate the obtained results, *Rickettsia*-positive samples were randomly selected for additional conventional and nested PCR assays using primers different from those used in the high-throughput microfluidic real-time PCR, as previously described [39].

Subsequently, selected *Rickettsia* and *Dermacentor* amplicons were sequenced by Eurofins MWG Operon (Ebersberg, Germany). Obtained nucleotide sequences were submitted to GenBank under accession numbers OR654148-50 (*Rickettsia*) and OR428530 and OR625082 (*Dermacentor*).

### 3.5. Phylogenetic Analysis

Sequences obtained in the current study were analyzed using Basic Local Alignment Search Tool (BLAST; https://blast.ncbi.nlm.nih.gov/Blast.cgi, accessed 1 October 2023) and aligned with sequences showing similarity using the MUSCLE algorithm in MEGA 11. Phylogenetic trees of *Rickettsia ompB* and *Dermacentor* ITS-2 were constructed using the Tamura 3-parameter model (T92) based on the lowest Bayesian information criterion and the corrected Akaike information criterion. The evolutionary history was inferred using maximum likelihood with a complete deletion option and bootstrap set to 1,000 and analyzed in MEGA 11 [41].

To determine the genetic diversity of *Dermacentor* specimens collected during this study, ITS-2 sequences shown in the phylogram were grouped into haplotypes (genotypes) using DnaSP software (Universitat de Barcelona, Spain). To show the genetic diversity of *D. reticulatus* depending on the geographical distribution, the median-joining network method available in POPArt software (University of Otago, New Zealand) was applied. In addition, to assess nucleotide differences between the analyzed ITS-2 sequences of *D. reticulatus*, evolutionary distances, represented as *p*-distance, were calculated using MEGA 11 [41].

### 3.6. Statistical Analysis

#### 3.6.1. Significance of Tick Population Structure and TBPs Prevalence

The Shapiro–Wilk test rejected the hypothesis of normal distribution of the analyzed data; therefore, the nonparametric Mann–Whitney *U* test was used to examine the significance of differences in the number of active female and male ticks. The Kruskal–Wallis test was used to analyze the significance of differences in the number of active ticks between the studied subregions. The *χ*^2^ test was used to analyze the significance of the prevalence of TBPs.

#### 3.6.2. Significance of Environmental Parameters on Tick Density

The relationship between altitude (a.s.l.) and tick density was examined using the rho-Spearman correlation. In addition, to determine the significance of selected environmental parameters on tick density across the Subcarpathian region, we employed random forest modeling. This method offers a comprehensive evaluation of variable significance without the prerequisite of feature selection [42]. The analysis was carried out using the “rfPermute” package in R [43], configured to generate 1,000 trees and perform 500 permutations. The variable importance was quantified using the percentage iIncrease in mean-squared error (%IncMSE), this metric measures the impact of each predictor variable on the predictive accuracy of the model. A higher %IncMSE value indicates a variable that is more critical for the model's predictive performance, as its alteration causes a larger degradation in the model's ability to accurately predict the outcome [42]. All analyses were executed within the R software environment version 4.3.1 (R Core Team, 2023) [43].

## 4. Results

### 4.1. Density and Range of the Occurrence of *Dermacentor reticulatus*

The study encompassed the collection of 1,556 *D. reticulatus* adults over the observation period. While females (878) outnumbered males (678), the observed differences were not statistically significant (*Z* = 0.4357, *p*=0.6599) (Table 1). Additionally, there was a nonsignificant predominance of tick counts in the autumn collection (881) compared to spring (675) (*Z* = −0.6173, *p*=0.5353).

The overall mean density of *D. reticulatus* across the entire Subcarpathian province was 3.5 ± 5.8 specimens/100 m^2^, displaying significant variation among the studied subregions (*H* = 18.6747, *p* < 0.0001) (Table 2).

**Figure 2:**
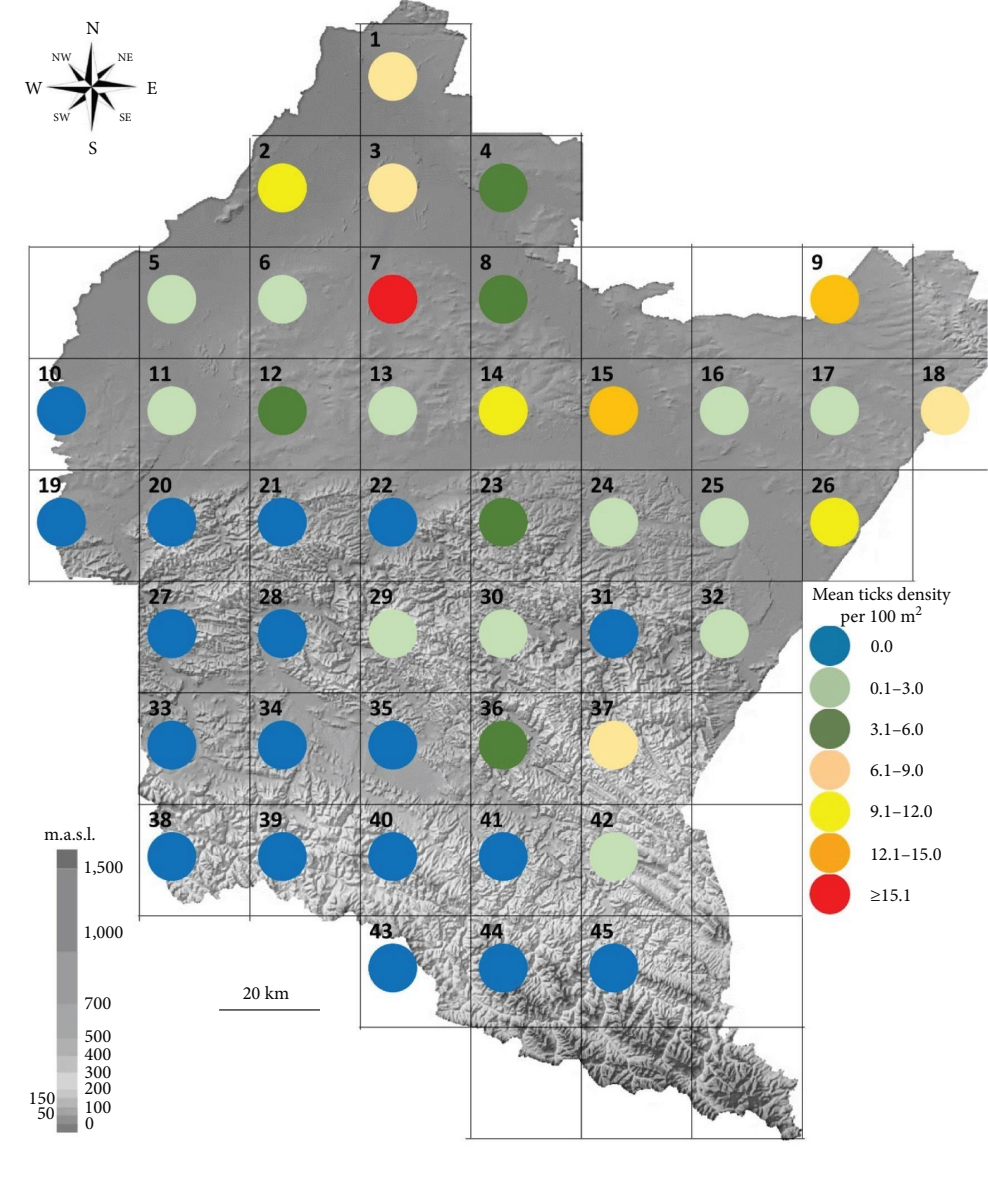
Mean density of *Dermacentor reticulatus* ticks in Subcarpathian province, Poland.

Notably, the highest density of *D. reticulatus* ticks was observed in the northern part of the region within the Sandomierz Basin, ranging from 0.0 to 30.8 specimens/100 m^2^, with a mean of 5.9 ± 7.1 specimens/100 m^2^. This particular subregion, excluding the western edges (sites 10 and 19), along with the eastern parts of the Central Beskids Foothills and the northern section of the Forested Beskids, constitutes the concentrated range of *D. reticulatus* in the Subcarpathian province (Figure 2 and Table 2). Within this compact range, the mean tick density was 5.8 ± 6.4 specimens/100 m^2^ (Table 1).

### 4.2. Impact of Environmental Parameters on Tick Density

With increasing elevation (m.a.s.l.), a decrease in the density of *D. reticulatus* ticks was observed. In the Central Beskids Foothills, its range was 1.0 ± 2.4 specimens/100 m^2^ on average, while within the mountain range (Central Beskids and Forested Beskids), the presence of *D. reticulatus* was confirmed at only one site, i.e., 42. The mean density of *D. reticulatus* in this area was 0.0–0.3 ± 0.0 specimens/100 m^2^ (Figure 2 and Table 2). A significant negative correlation between elevation and the density of *D. reticulatus* ticks was confirmed (*r*_s_ = −0.5031, *p*=0.0004).

**Figure 3:**
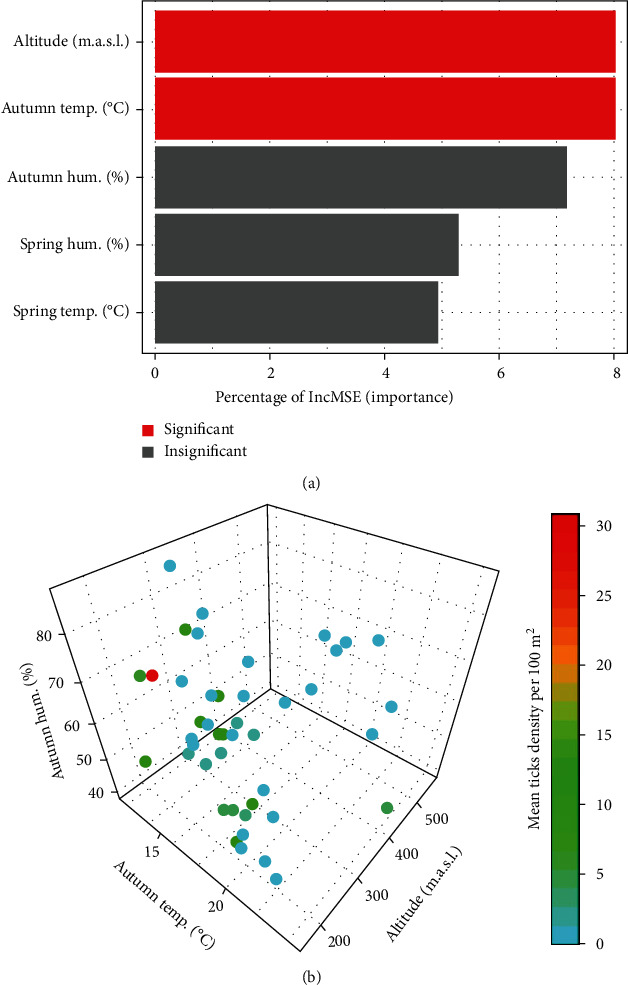
Multifactorial analysis of environmental parameters influencing tick density per 100 m^2^ utilizing a random forest model: (a) relative importance of each environmental parameter, quantified by the percentage increase in mean-squared error (%IncMSE), with altitude and autumn temperature identified as the significant factors; (b) 3D visualization of the data, where the distribution of tick densities is plotted against variations in altitude, autumn temperature (°C), and autumn humidity (%), and the color gradient denotes the mean tick density per 100 m^2^.

In our effort to identify the environmental determinants of tick density in the Subcarpathian region, random forest modeling emerged as a reliable screening method. It effectively ranked the significance of various variables, including altitude, as well as temperature and humidity data obtained during both the spring and autumn seasons. The resulting hierarchy of factors, depicted in Figure 3, identified altitude as the most influential parameter, followed by autumn temperature and humidity. These findings, represented through the 3D scatterplot (Figure 3), underscore the varying impact of environmental conditions on tick distribution.

### 4.3. Prevalence and Phylogeny of TBPs in *Dermacentor reticulatus*

**Figure 4:**
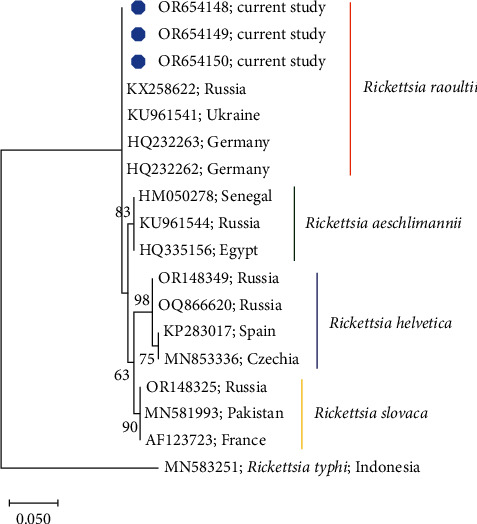
Phylogeny of spotted fever group *Rickettsia* based on *ompB* gene. The evolutionary history was inferred by using the maximum-likelihood method and the Tamura 3-parameter model. The analysis contains sequences identified in the current study (marked with blue dot) and GenBank sequences. Accession numbers of sequences and country of origin are given. Bootstrap values are represented as percentage of internal branches (1,000 replicates), and values lower than 60 are hidden. The tree is drawn to scale, with branch lengths measured in the number of substitutions per site. *Rickettsia typhi* sequence MN583151 was used to root the tree.

We found that *D. reticulatus* ticks were only infected with *Rickettsiales*, while there was no evidence of infection with *Borrelia* spp. and *B. canis* (Table 3). The analysis of the *ompB* gene sequencing revealed that the *Rickettsia* spp. samples were 100% identical to *R. raoultii* and clustered together with previously reported from Ukraine, Germany, and Russia (Figure 4). *R. raoultii*-infected ticks were found in all tested sites, but the distribution was not uniform. The highest number of infected ticks was confirmed in the northern part of the region (site 2, up to 84.21%), while the lowest was in the northeastern part (site 9, up to 20.00%). In contrast, in the Central Beskids Foothills area, the share of infected ticks was also high, up to 71.43% (Table 3). The study sites varied significantly in the prevalence of *R. raoultii* in *D. reticulatus* (*χ*^2^ = 40.089, *p* < 0.0001).

Only ticks collected from the northern part of the Sandomierz Basin (site 2) were found to be infected with *R. helvetica* and *A. phagocytophilum*. The prevalence of infection with these pathogens was 9.52% and 4.76%, respectively.

### 4.4. Genetic Diversity of *Dermacentor reticulatus*

**Figure 5:**
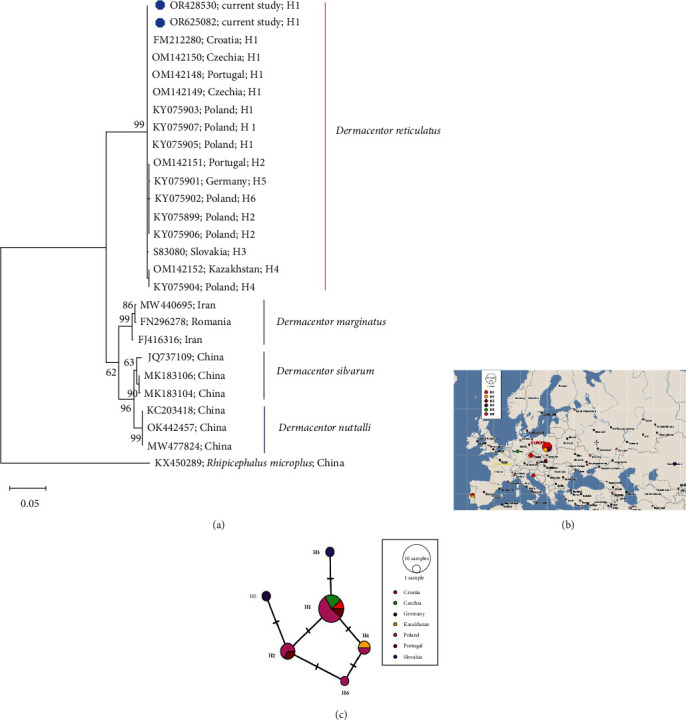
Genetic diversity of *Dermacentor reticulatus*: (a) phylogeny of *Dermacentor* spp. based on ITS-2. The evolutionary history was inferred by using the maximum-likelihood method and the Tamura 3-parameter model. The analysis contains sequences identified in the current study (marked with blue dot) and GenBank sequences. Accession numbers of sequences and country of origin are given. Bootstrap values are represented as percentage of internal branches (1,000 replicates), and values lower than 60 are hidden. The tree is drawn to scale, with branch lengths measured in the number of substitutions per site. *Rhipicephalus microplus* sequence KX450289 was used to root the tree; H—haplotype. (b) Geographical distribution of *D. reticulatus* haplotypes based on sequence analysis ITS-2 obtained in the current study and sequences available in GenBank. (c) Network analysis of geographical distribution of *D. reticulatus* haplotypes based on analysis of ITS-2 sequence obtained in the current study and sequences available in GenBank. The diagonal lines indicate the number of mutations between haplotypes.

The phylogenetic analysis of ITS-2 sequences obtained in this study confirms the species' affiliation with *D. reticulatus* (Figure 5); these sequences showed a lack of genetic variation (Table 4). Examining *D. reticulatus* sequences from GenBank having similarities with those from the current study, revealed genetic variation with six haplotypes. Most of these sequences originate from Europe, with one haplotype recorded in Kazakhstan, Asia. The dominant haplotype among *D. reticulatus* was H1, consistent with its identification in the sequences obtained in this study. Furthermore, the research indicates a high genetic diversity of *D. reticulatus* populations in Poland, where four out of six haplotypes were identified. This contrasts with the low genetic diversity in the studied region, where only one haplotype was identified (Figure 5 and Table 4).

## 5. Discussion

In the current study, we confirmed the presence of *D. reticulatus* ticks in the Subcarpathian region; however, the compact range of its occurrence is limited only to the foothills and lowlands, while mountainous areas should be considered free of this tick species (Figure 2, Tables 1 and 2). Similar relationships were observed in our previous study on tick occurrence and activity in the region of the Western Carpathian Mountains [44], during which we showed that the only tick species collected from vegetation was *Ixodes ricinus*. On the other hand, the occurrence of *D. reticulatus* in Western Carpathian was accidental and limited only to specimens feeding on hosts. However, the occurrence of *D. reticulatus* in mountain habitats has been confirmed in other European countries [15, 17, 19, 22]. On the other hand, the studied region is located in a temperate climate zone [33] with various features, and the prevailing weather conditions are within the tolerance range of *D. reticulatus* [1, 4, 18]. Nevertheless, analysis of the obtained results allowed us to identify altitude as the most influential parameter, followed by temperature (Figure 3). The impact of temperature as a significant factor limiting the activity and/or occurrence of *D. reticulatus* has been confirmed repeatedly in previous studies [45, 46].

The ecological type of habitat/dominant vegetation and availability of potential hosts are relevant factors influencing the size of local *D. reticulatus* populations [1]. In our current study, we have shown that the highest density of *D. reticulatus* in the Subcarpathian province is found in the subregion with the lowest forest cover (i.e., Sandomierz Basin) [33] (Figure 2, Tables 1, and 2). A similar pattern was also observed in other regions of Poland [24, 47]. The preferred hosts of *D. reticulatus* adults are mainly medium-sized game animals, while juvenile stages feed on small rodents [48]. In our previous studies conducted in Subcarpathian, we confirmed the presence of animals that could serve as potential hosts for *D. reticulatus*, i.e., rodents *Apodemus agrarius*, *A. flavicollis*, *Microtus* spp., *Myodes glareolus*, and Artiodactyla, i.e., *Capreolus capreolus* [44]. Considering the environmental conditions prevailing in the study area and the ecological preferences of *D. reticulatus*, it allows the assumption that the area of the Subcarpathian is currently a limit of the compact geographical range of *D. reticulatus* in southeastern Poland.

The results of our study indicate that the region of southeastern Poland is characterized by a moderate density of *D. reticulatus* (an average of 5.8 ± 6.4 specimens/100 m^2^ in the area of the compact range) (Table 1) compared to the rest of the country [27, 28]. Moreover, the density of *D. reticulatus* in the Subcarpathian province is on average 16.6 times lower than in eastern Poland (Lublin province) [24]. Noteworthy is the fact that in areas bordering the two provinces, the density of *D. reticulatus* ticks is comparable, ranging from 25.0 to 34.0 in Lublin province and 14.8–30.8 in Subcarpathian province [24] (Figure 2 and Table 1). This supports the above-mentioned hypothesis about the current range limit of *D. reticulatus* in the studied region. For this reason, further investigations to monitor the spread of this species in the Subcarpathian are crucial.

The present results show that *D. reticulatus* tick is an important vector and reservoir of Rickettsiales in the studied region (Table 3). The phylogenetic analysis allowed us to conclude that the dominant pathogen detected in *D. reticulatus* is *R. raoultii* (up to 84.21%) (Table 3 and Figure 4). Also, our previous study from eastern Poland confirmed the high prevalence of *Rickettsia* spp. infection in *D. reticulatus* reaching 91.70% [38]. Meanwhile, on a national scale, there is a clear variation of *Rickettsia* spp. prevalence in *D. reticulatus* ticks depending on the region. In northeastern Poland, up to 30.20% of specimens of *D. reticulatus* ticks are infected with this pathogen [49]. At the same time, in this region, high levels of *Rickettsia*-reactive IgG were observed in foresters (51.22%) and farmers (26.83%) [50]. Of particular interest are studies reporting the high prevalence of RSFG agents detected in *D. reticulatus* ticks removed from human skin [51]. The results of the mentioned study confirmed the high prevalence of *Rickettsia* spp. (50.00%), including *R. aeschlimannii*. It is also noteworthy that 13.3% of the surveyed patients presented localized skin lesions at the tick bite site and flu-like symptoms [51].

The high prevalence of *Rickettsia* spp. in *D. reticulatus* (up to 74.4%) has been confirmed in other countries of the region, including Czechia, Slovakia, and Hungary [21, 52, 53]. Also, *D. reticulatus* ticks frequently transmit *R. slovaca* and *R. helvetica* [1]. The presence of this pathogen was also confirmed in the current study (up to 9.52%) (Table 3). Recently, *R. aeschlimannii* is also more frequently detected in *D. reticulatus* [38, 51]. Moreover, in our study, we confirmed infection of *D. reticulatus* by *A. phagocytophilum* (4.76%) (Table 3), which is a less frequently detected pathogen in this species [54, 55].

*D. reticulatus* ticks play an important role in the transmission of *Babesia* spp.; however, in the current study, we did not confirm the presence of genetic material of this protozoan in the examined specimens (Table 3), in contrast to the results of our earlier study from eastern Poland where up to 12.50% of ticks were infected [38]. A similar prevalence of *Babesia* spp. (9.20%) was reported from northeastern Poland [56]. Other reports from Poland show a varying prevalence of *B. canis* depending on the endemic nature of the studied area, i.e., 6.10% in the eastern endemic zone and 3.30% in the eastern expansion zone [10]. Considering the above, we believe that the absence of *D. reticulatus* ticks infected with *Babesia* spp. may indicate a limited occurrence of this protozoan in the studied region.

In our study, we also did not detect the presence of *Borrelia* spp. in *D. reticulatus* ticks (Table 3). Similarly, during our previous 3-year study, we did not observe infection with spirochetes of this species in ticks collected in forest habitats [38]. Nevertheless, *Borrelia* spp. infections in *D. reticulatus* collected from vegetation were reported in other studies, but the prevalence of infection with this pathogen was low (0.25%–0.30%) [45, 56]. In contrast, the results of another study show that 12.70% of *D. reticulatus* ticks removed from human skin tested positive for *Borrelia* presence [57].

It is believed that the changes in the range of *D. reticulatus* ticks observed nowadays are the result of both glaciations, the Little Ice Age and the migration of large mammals—the main hosts of this species [58]. These changes can be reflected in the pattern of genetic variation among local populations of *D. reticulatus*. Our analysis of ITS-2 shows the belonging of *D. reticulatus* occurring in Subcarpathian to the same haplotype H1 (Figure 5). At the same time, analyzed ITS-2 sequences showed similarity with other sequences from Poland and countries of the region, i.e., Czechia, Croatia, and geographically distant Portugal. In total, the analysis of genetic diversity allowed the identification of six haplotypes (Figure 5).

Against this background, the case of Poland is particularly interesting, where, according to our analysis, four haplotypes of ITS-2 were identified (Figure 5). According to Paulauskas et al. [59], Poland is a contact zone of two clusters of *D. reticulatus* populations, i.e., one from the west and another from the east of the range. The multiplicity of haplotypes present in Poland, as well as the changes in the range of *D. reticulatus* in the country evidenced in recent years, may suggest gene flow between both mentioned clusters [27, 60, 61]. In addition, microsatellite markers have demonstrated increased gene flow for ticks with a variety of feeding strategies [62, 63]; therefore, it can be concluded that the wide spectrum of potential hosts of *D. reticulatus* favors genetic divergence.

## 6. Conclusions

Our findings contribute to understanding the ecological factors influencing *D. reticulatus* populations, particularly in the Subcarpathian region which can be considered as the limit area of the compact range of these ticks in Poland. The study underscores the importance of considering environmental variables, such as air temperature and altitude, in predicting tick distribution. In the study area, adult *D. reticulatus* specimens exhibit a limited range of vectored pathogens (specifically, only Rickettsiales were detected) and demonstrate a low level of genetic variation. Additionally, the prevalence of *R. raoultii* highlights the public health relevance of *D. reticulatus* as a vector of TBPs in the studied area. Ongoing monitoring and further investigations are crucial to assess the potential spread of this tick species and its associated pathogens in the Subcarpathian region.

## Figures and Tables

**Table 1:** Density of *Dermacentor reticulatus* ticks in Subcarpathian region.

Plot number	Geographical coordinates	Altitude (m.a.s.l.)	Number of collected ticks per 500 m^2^													Mean ticks density per 100 m^2^		
			Spring					Autumn					Mean number of collected ticks per 500 m^2^					
			Weather parameters		Number of collected ticks			Weather parameters		Number of collected ticks								
			T (°C)	H (%)	F	M	F + M	T (°C)	H (%)	F	M	F + M	F	M	F + M	F	M	F + M
1	50.757; 22.080	219	17.0	61.0	11	6	17	18.5	67.9	18	28	46	14.5	17	31.5	2.9	3.4	6.3
2	50.585; 21.848	149	13.6	68.1	33	20	53	15.4	60.1	31	19	50	32	19.5	51.5	6.4	3.9	10.3
3	50.603; 22.137	190	15.7	64.5	11	6	17	20.2	49.9	32	13	45	21.5	9.5	31	4.3	1.9	6.2
4	50.551; 22.395	226	18.2	51.9	3	3	6	19.3	48.3	17	12	29	10	7.5	17.5	2.0	1.5	3.5
5	50.438; 21.585	158	16.4	66.7	0	4	4	23.0	68.0	2	6	8	1	5	6	0.2	1.0	1.2
6	50.403; 21.755	187	17.0	69.0	2	3	5	20.1	46.0	0	0	0	1	1.5	2.5	0.2	0.3	0.5
7	50.378; 22.122	212	17.3	62.7	96	76	172	14.3	72.6	69	67	136	82.5	71.5	154	16.5	14.3	30.8
8	50.266; 22.387	201	21.0	63.3	6	6	12	16.6	60.3	15	12	27	10.5	9	19.5	2.1	1.8	3.9
9	50.342; 23.356	273	20.8	60.0	44	16	60	16.4	67.7	46	42	88	45	29	74	9.0	5.8	14.8
10	50.210; 21.419	189	18.1	57.9	0	0	0	17.5	66.0	0	0	0	0	0	0	0.0	0.0	0.0
11	50.183; 21.606	190	17.8	59.0	2	0	2	19.7	46.0	4	5	9	3	2.5	5.5	0.6	0.5	1.1
12	50.218; 21.747	202	14.0	65.3	6	6	12	18.6	50.6	18	24	42	12	15	27	2.4	3.0	5.4
13	50.256; 22.138	256	16.2	61.4	5	2	7	14.6	54.5	3	10	13	4	6	10	0.8	1.2	2.0
14	50.259; 22.321	248	14.4	59.9	17	8	25	11.3	64.5	42	38	80	29.5	23	52.5	5.9	4.6	10.5
15	50.252; 22.708	231	18.5	38.0	59	42	101	16.6	65.4	24	23	47	41.5	32.5	74	8.3	6.5	14.8
16	50.222; 23.004	260	21.0	38.9	3	3	6	16.7	55.2	6	3	9	4.5	3	7.5	0.9	0.6	1.5
17	50.186; 23.191	204	20.0	51.5	5	6	11	17.6	59.8	5	3	8	5	4.5	9.5	1.0	0.9	1.9
18	50.150; 23.280	248	18.9	50.0	9	15	24	19.1	48.5	19	18	37	14	16.5	30.5	2.8	3.3	6.1
19	50.256; 22.138	225	18.2	59.9	0	0	0	20.5	37.6	0	0	0	0	0	0	0.0	0.0	0.0
20	50.022; 21.427	336	17.7	55.0	0	0	0	15.1	49.7	0	0	0	0	0	0	0.0	0.0	0.0
21	50.062; 21.718	227	16.8	64.8	0	0	0	17.2	66.6	0	0	0	0	0	0	0.0	0.0	0.0
22	49.989; 22.028	251	17.0	70.0	0	0	0	16.8	70.5	0	0	0	0	0	0	0.0	0.0	0.0
23	49.984; 22.312	360	14.3	61.4	14	6	20	24.7	51.9	17	6	23	15.5	6	21.5	3.1	1.2	4.3
24	49.965; 22.608	271	21.5	48.2	1	0	1	19.3	50.6	0	0	0	0.5	0	0.5	0.1	0.0	0.1
25	50.007; 22.874	209	16.4	61.4	9	3	12	19.0	50.9	13	5	18	11	4	15	2.2	0.8	3.0
26	50.017; 23.119	204	19.9	49.9	17	27	44	18.8	70.1	34	25	59	25.5	26	51.5	5.1	5.2	10.3
27	49.807; 21.581	295	11.8	77.1	0	0	0	15.2	81.8	0	0	0	0	0	0	0.0	0.0	0.0
28	49.863; 21.755	295	10.0	77.5	0	0	0	13.4	88.2	0	0	0	0	0	0	0.0	0.0	0.0
29	49.871; 22.050	277	7.7	78.9	5	5	10	15.4	79.1	4	1	5	4.5	3	7.5	0.9	0.6	1.5
30	49.826; 22.353	234	11.4	69.2	7	3	10	15.5	72.2	6	5	11	6.5	4	10.5	1.3	0.8	2.1
31	49.871; 22.693	277	20.4	40.2	0	0	0	17.5	63.3	0	0	0	0	0	0	0.0	0.0	0.0
32	49.799; 22.925	187	20.0	50.9	1	0	1	22.3	44.6	6	2	8	3.5	1	4.5	0.7	0.2	0.9
33	49.667; 21.478	291	12.8	62.7	0	0	0	18.0	77.0	0	0	0	0	0	0	0.0	0.0	0.0
34	49.599; 21.704	357	13.0	67.5	0	0	0	18.4	64.0	0	0	0	0	0	0	0.0	0.0	0.0
35	49.667; 21.993	310	14.0	74.0	0	0	0	17.0	66.2	0	0	0	0	0	0	0.0	0.0	0.0
36	49.702; 22.213	256	10.2	79.5	2	0	2	19.2	66.2	21	12	33	11.5	6	17.5	2.3	1.2	3.5
37	49.640; 22.671	308	18.6	65.6	28	13	41	13.3	75.0	26	19	45	27	16	43	5.4	3.2	8.6
38	49.500; 21.594	465	12.7	70.3	0	0	0	18.2	72.2	0	0	0	0	0	0	0.0	0.0	0.0
39	49.429; 21.856	513	14.4	63.2	0	0	0	18.0	65.6	0	0	0	0	0	0	0.0	0.0	0.0
40	49.410; 22.140	427	14.1	62.0	0	0	0	18.4	62.2	0	0	0	0	0	0	0.0	0.0	0.0
41	49.457; 22.278	486	12.3	64.6	0	0	0	21.4	51.5	0	0	0	0	0	0	0.0	0.0	0.0
42	49.451; 22.593	474	12.8	64.8	0	0	0	22.4	62.2	4	1	5	2	0.5	2.5	0.4	0.1	0.5
43	49.317; 22.061	522	12.1	67.0	0	0	0	18.5	68.0	0	0	0	0	0	0	0.0	0.0	0.0
44	49.344; 22.264	538	13.9	70.0	0	0	0	18.1	66.1	0	0	0	0	0	0	0.0	0.0	0.0
45	49.340; 22.672	578	11.3	75.8	0	0	0	19.5	66.6	0	0	0	0	0	0	0.0	0.0	0.0
Total/mean ± SD	—	—	—	—	396	279	675	—	—	482	399	881	16.3^*∗*^	12.6^*∗*^	28.9^*∗*^	3.3 ± 3.5^*∗*^	2.5 ± 2.9^*∗*^	5.8 ± 6.4^*∗*^

T, temperature; H, humidity; SD, standard deviation;  ^*∗*^mean values calculated only on data from plots where the presence of ticks was confirmed.

**Table 2:** Mean density of *Dermacentor reticulatus* ticks per 100 m^2^ in particular subregions.

Subregions	Tick collection sites	Sex	Mean density	SD	Min.	Max.
Sandomierz Basin	1–19, 24–26, 32	F	3.3	3.9	0.0	16.5
		M	2.6	3.2	0.0	14.3
		F + M	5.9	7.1	0.0	30.8

Central Beskids Foothills	20–22, 27–31, 33–35, 37	F	0.6	1.5	0.0	5.4
		M	0.4	0.9	0.0	3.2
		F + M	1.0	2.4	0.0	8.6

Central Beskids	38, 39, 43	F	0.0	0.0	0.0	0.0
		M	0.0	0.0	0.0	0.0
		F + M	0.0	0.0	0.0	0.0

Forested Beskids	41, 42, 44, 45	F	0.2	0.0	0.0	0.4
		M	0.1	0.0	0.0	0.1
		F + M	0.3	0.0	0.0	0.5

All studied subregions	1–45	F	2.0	3.2	0.0	16.5
		M	1.5	2.6	0.0	14.3
		F + M	3.5	5.8	0.0	30.8

F, females; M, males; SD, standard deviation; Min., minimum; Max., maximum.

**Table 3:** Prevalence of tick-borne pathogens detected in *Dermacentor reticulatus* in Subcarpathian region.

Subregions	Tick collection site	Tick stages	Tick-borne pathogens/number of positive samples and percentage rate (%)				
			*Borrelia* spp.	*Rickettsia raoultii*	*Rickettsia helvetica*	*Anaplasma phagocytophilum*	*Babesia canis*
Sandomierz Basin	2	Females *n* = 21	0 (0.00)	13 (61.90)	2 (9.52)	1 (4.76)	0 (0.00)
		Males *n* = 19	0 (0.00)	16 (84.21)	0 (0.00)	0 (0.00)	0 (0.00)
	9	Females *n* = 20	0 (0.00)	9 (45.00)	0 (0.00)	0 (0.00)	0 (0.00)
		Males *n* = 20	0 (0.00)	4 (20.00)	0 (0.00)	0 (0.00)	0 (0.00)

Central Beskids Foothills	37	Females *n* = 21	0 (0.00)	15 (71.43)	0 (0.00)	0 (0.00)	0 (0.00)
		Males *n* = 19	0 (0.00)	12 (63.16)	0 (0.00)	0 (0.00)	0 (0.00)

All studied subregions		Females *n* = 62	0 (0.00)	37 (59.68)	2 (3.23)	1 (1.61)	0 (0.00)
		Males *n* = 58	0 (0.00)	32 (55.17)	0 (0.00)	0 (0.00)	0 (0.00)

*n*, number of tested specimens.

**Table 4:** Evolutionary distances between the pairs of analyzed *Dermacentor reticulatus* ITS-2 sequences (calculated as *p*-distance values); sets of sequences correspond to Figure 5.

Accession numbers and regions of origin		Current study		Poland							Europe							Asia
		OR428530	OR625082	KY075906	KY075903	KY075904	KY075907	KY075905	KY075902	KY075899	FM212280	OM142150	OM142149	S83080	OM142148	OM142151	KY075901	OM142152
Current study	OR428530	—	—	—	—	—	—	—	—	—	—	—	—	—	—	—	—	—
	OR625082	0.00000	—	—	—	—	—	—	—	—	—	—	—	—	—	—	—	—

Poland	KY075906	0.00176	0.00176	—	—	—	—	—	—	—	—	—	—	—	—	—	—	—
	KY075903	0.00000	0.00000	0.00176	—	—	—	—	—	—	—	—	—	—	—	—	—	—
	KY075904	0.00176	0.00176	0.00352	0.00176	—	—	—	—	—	—	—	—	—	—	—	—	—
	KY075907	0.00000	0.00000	0.00176	0.00000	0.00176	—	—	—	—	—	—	—	—	—	—	—	—
	KY075905	0.00000	0.00000	0.00176	0.00000	0.00176	0.00000	—	—	—	—	—	—	—	—	—	—	—
	KY075902	0.00352	0.00352	0.00176	0.00352	0.00176	0.00352	0.00352	—	—	—	—	—	—	—	—	—	—
	KY075899	0.00176	0.00176	0.00000	0.00176	0.00352	0.00176	0.00176	0.00176	—	—	—	—	—	—	—	—	—

Europe	FM212280	0.00000	0.00000	0.00176	0.00000	0.00176	0.00000	0.00000	0.00352	0.00176	—	—	—	—	—	—	—	—
	OM142150	0.00000	0.00000	0.00176	0.00000	0.00176	0.00000	0.00000	0.00352	0.00176	0.00000	—	—	—	—	—	—	—
	OM142149	0.00000	0.00000	0.00176	0.00000	0.00176	0.00000	0.00000	0.00352	0.00176	0.00000	0.00000	—	—	—	—	—	—
	S83080	0.00176	0.00176	0.00352	0.00176	0.00352	0.00176	0.00176	0.00529	0.00352	0.00176	0.00176	0.00176	—	—	—	—	—
	OM142148	0.00000	0.00000	0.00176	0.00000	0.00176	0.00000	0.00000	0.00352	0.00176	0.00000	0.00000	0.00000	0.00176	—	—	—	—
	OM142151	0.00176	0.00176	0.00000	0.00176	0.00352	0.00176	0.00176	0.00176	0.00000	0.00176	0.00176	0.00176	0.00352	0.00176	—	—	—
	KY075901	0.00352	0.00352	0.00176	0.00352	0.00529	0.00352	0.00352	0.00352	0.00176	0.00352	0.00352	0.00352	0.00529	0.00352	0.00176	—	—

Asia	OM142152	0.00176	0.00176	0.00352	0.00176	0.00000	0.00176	0.00176	0.00176	0.00352	0.00176	0.00176	0.00176	0.00352	0.00176	0.00352	0.00529	—

## Data Availability

The data used to support the findings of this study are available from the corresponding author upon request.

## Supplementary Materials

Supplementary Materials Table S1: list of primers used in the current study for microfluidic real-time PCR, based on Michelet et al. [40].
