# Supplementary material for: Spatial Distribution and Pathogen Profile of Dermacentor reticulatus Ticks in Southeastern Poland: A Genetic and Environmental Analysis
Source: Transbound Emerg Dis. 2024 Apr 24;2024:5458278. doi: 10.1155/2024/5458278 (PMC12017008; doi:10.1155/2024/5458278)
Supplement: Supplementary Materials — Table S1: list of primers used in the current study for microfluidic real-time PCR, based on Michelet et al. [40]. [file 5458278.f1.docx]

Supplementarny Table 1. List of primers used in the current study for microfluidic real-time PCR, based on Michelet et al.

| **Pathogen** | **Target Gene** | **Primers** | **Length (bp)** |
| --- | --- | --- | --- |
| *Borreliella* spp. | *23S rRNA* | F-GAGTCTTAAAAGGGCGATTTAGT,  R-CTTCAGCCTGGCCATAAATAG | 73 |
| *Borreliella. burgdorferi* s.s. | *rpoB* | F-GCTTACTCACAAAAGGCGTCTT,  R-GCACATCTCTTACTTCAAATCCT | 83 |
| *Borreliella. garinii* | *rpoB* | F-TGGCCGAACTTACCCACAAAA,  R-ACATCTCTTACTTCAAATCCTGC | 88 |
| *Borreliella valaisiana* | *ospA* | F-ACTCACAAATGACAGATGCTGAA,  R-GCTTGCTTAAAGTAACAGTACCT | 135 |
| *Borreliella afzelii* | *fla* | F-GGAGCAAATCAAGATGAAGCAAT,  R-TGAGCACCCTCTTGAACAGG | 116 |
| *Borreliella spielmanii* | *fla* | F-ATCTATTTTCTGGTGAGGGAGC,  R-TCCTTCTTGTTGAGCACCTTC | 71 |
| *Borrelia* *miyamotoi* | *glpQ* | F-CACGACCCAGAAATTGACACA,  R-GTGTGAAGTCAGTGGCGTAAT | 94 |
| *Ehrlichia* spp. | *16S rRNA* | F-GCAACGCGAAAAACCTTACCA, R-AGCCATGCAGCACCTGTGT | 98 |
| *Rickettsia* spp. | *gltA* | F-GTCGCAAATGTTCACGGTACTT R-TCTTCGTGCATTTCTTTCCATTG | 78 |
| *Rickettsia helvetica* | *ITS* | F-TTTGAAGGAGACACGGAACACA, R-TCCGGTACTCAAATCCTCACGTA | 65 |
| *Anaplasma phagocytophilum* | *msp2* | F-GCTATGGAAGGCAGTGTTGG R-GTCTTGAAGCGCTCGTAACC | 77 |
| *Babesia canis* | *18S rRNA* | F-TGGCCGTTCTTAGTTGGTGG R-AGAAGCAACCGGAAACTCAAATA | 104 |
| *Babesia divergens* | *hsp70* | F-CTCATTGGTGACGCCGCTA,  R-CTCCTCCCGATAAGCCTCTT | 83 |
| *Babesia venatorum* | *18S rRNA* | F-AACCTGGTTGATCCTGCCAGT, R- GCTTGATCCTTCTGCAGGTTCACCTAC | 31 |
| *Dermacentor reticulatus* | *ITS2* | F- AACCCTTTTCCGCTCCGTC,  R-TTTTGCAGAGCTCGACGTAC | 83 |
| *Ixodes ricinus* | *ITS2* | F-TGCGTTGCGTCTTCTCTTGTT,  R-ATCTCCACGCACCGACGT | 77 |
| *Escherichia coli* | *eae* | F-CATTGATCAGGATTTTTCTGGTGAT R-CTCATGCGGAAATAGCCGTTA | 102 |

Michelet L, Delannoy S, Devillers E et al (2014) High-throughput screening of tick-borne pathogens in Europe. Front Cell Inf Microbiol 4:103. <https://doi.org/10.3389/fcimb.2014.00103>
